# “It’s All about Calculations—But There Are No Definite Results”—Parental Adaptation and Coping during the First Month following Their Child’s Diabetes Diagnosis

**DOI:** 10.3390/healthcare11020280

**Published:** 2023-01-16

**Authors:** Louise Norman Jespersen, Kristine Zoega Mikkelsen, Dan Grabowski

**Affiliations:** Department of Health Promotion Research, Diabetes Management, Copenhagen University Hospital, Steno Diabetes Center Copenhagen, Borgmester Ib Juuls Vej 83, 2730 Herlev, Denmark

**Keywords:** newly diagnosed, diabetes, children, adolescents, families, parents, adaptation, coping

## Abstract

Diabetes-related habits established during the first few years after diagnosis are difficult to change. Therefore, the initial period after a child has been diagnosed with diabetes holds a unique potential for early interventions to adjust adverse patterns of diabetes self-management before they become firmly established. Family functioning is strongly related to glycemic levels, and attending to parents’ needs at the time of diagnosis could therefore reduce psychological distress and improve glycemic levels in their children. This study aims to investigate parental adaptation to and coping with their child’s diabetes diagnosis during the first month post-diagnosis. Twenty interviews with parents of children (0–18 years) with newly diagnosed type 1 diabetes were conducted and analyzed using systematic text condensation. Four themes were identified: (1) Removal of the safety net, when families experience that they are now on their own; (2) Hit by the realities, when parents realize the impact of living with diabetes; (3) Hang in there, when families mobilize resources to get them through a tough period; and (4) Toward a new normal, when parents begin to reestablish their life with diabetes in mind. This study generated unique insights into early parental adaptation and coping after their child was diagnosed with diabetes.

## 1. Introduction

When a child is diagnosed with type 1 diabetes, it is most likely to happen suddenly, with little time to mentally prepare for a disruption that will alter the entire family’s way of life. Guidelines suggest that the child or parents, already from the day that the child is diagnosed, should be informed that they will become the central members of the care team [1]. While potentially dealing with shock and grief [1,2], the family suddenly needs to acquire a large amount of new knowledge that is crucial to mastering the comprehensive self-management regimen of childhood diabetes.

It is well known that diabetes-related habits and coping strategies established within a family during the first few years after diagnosis are difficult to change [3]. Therefore, the initial period after a child has been diagnosed with diabetes holds a unique potential for early interventions to adjust any adverse patterns of diabetes self-management before they become firmly established [3]. Given the comprehensiveness of the self-management regimen, treatment outcomes depend largely on how the children and their parents implement complex diabetes management in the various settings of everyday life. Moreover, one study concluded that the level and intensity of family involvement are better predictors of metabolic regulation than age, gender, and insulin treatment regimen [4].

However, having to constantly deal with disease management tasks in daycare, school, at home, and in their leisure time in various settings of everyday life places a heavy burden on the children and their parents. A review from 2012 indicated that parents find that their child’s diabetes contributes to significant disruption in their family [5], and parents have been found to experience enhanced levels of psychosocial stressors, such as anxiety, stress, and depression [6,7,8]. As a consequence of managing their child’s diabetes, parents of children with type 1 diabetes have also been found to experience how the combination of exhaustion, stress, grief, and a feeling of powerlessness result in diabetes burnout [9]. A study on parental adaptation to their child being diagnosed with type 1 diabetes showed that parents interviewed six months post-diagnosis felt distressed by their child’s diagnosis [8]. Even 5–6 years after the child’s diagnosis, parents who, at the time of diagnosis, felt high levels of uncertainty related to their child’s illness were more likely to report enhanced psychological distress [10]. Thus, the authors suggested that attending to parents’ feelings of uncertainty at the time of diagnosis could reduce future psychological distress.

Furthermore, family functioning has been found to strongly relate to glycemic levels [11,12]. A recent study of 60 children (10–18 years old) revealed that low levels of resilience and high levels of diabetes distress three months post-diagnosis impacted HbA1c levels up to three years later. The authors suggested that early interventions could change this impact by lowering the level of distess [12].

Although there is agreement that early intervention may play a key role in reducing psychological distress in parents of children with diabetes, little is known about how parents adapt to the diagnosis during the first few weeks following diagnosis. Our in-depth qualitative data, collected within a month after the families were discharged, allow us to address a gap in the literature by aiming to investigate and provide novel insights into parental coping with and adaptation to their child’s diabetes diagnosis during the first month post-diagnosis.

## 2. Materials and Methods

The study was designed as a longitudinal study—the ONSET Study. Qualitative interviews with the parents of a child with diabetes were conducted at three different points in time. This article derives from the first interview, which was conducted less than four weeks after the diagnosis.

### 2.1. Recruitment and Participants

The target group for the present study was parents of children (0–18 years) with newly diagnosed type 1 diabetes. Parents were recruited during the hospitalization following the diabetes diagnosis. Health Care Professionals (HCPs) initiated the contact by asking the parents for permission for the researchers to contact them by telephone when they had been discharged. Parents who agreed to be contacted shared their contact details with the HCP, who then passed on the information to the researchers. In total, 25 families were contacted by the researchers; 20 of them agreed to participate and were thus enrolled in the study.

The included families had children in the age range of 1–17 years. Six children were preschoolers, and three were teenagers. Half of the children were girls. Further characteristics of the included families are shown in Table 1.

### 2.2. Data Collection

Most of the interviews (75%) were conducted within three weeks after the families had been discharged from the hospital; 96% were conducted within four weeks. Interviews were conducted between October 2021 and June 2022.

Whenever possible, we encouraged both parents and the child to participate in the interview. In 13 interviews, both parents participated. Many parents explained that, since the time of the child’s diagnosis, there had been an intense focus on the child’s diabetes and that the children were fed up with talking about diabetes and did not want to participate in the interview. Some children did not participate at all, and nearly all participating children took the opportunity to withdraw from the interview.

All interviews were semi-structured and followed the same interview guide. Author KZM conducted all interviews and was assisted by LNJ in half of the interviews. In the remaining interviews, KZM was assisted by a student assistant. Most interviews lasted about an hour (range 41–87 min) and were conducted in the families’ own homes at a time of their choosing.

All interviews were audio recorded (with consent) and transcribed verbatim by KZM and two student assistants. Before the interviews, written informed consent was obtained from all parents. The present study complies with the ethical guidelines of the Declaration of Helsinki [13] and was approved by the Danish Regional Data Protection Agency. Registration number: P-2019-660. According to the Danish National Ethical Committees, interview studies do not require ethical approval.

### 2.3. Analysis

The data were analyzed stepwise using systematic text condensation, as described by Malterud (2012) [14]. Each of the analytical steps in the systematic text condensation was performed repeatedly during the analysis. This stepwise approach led to several changes and modifications before agreement on the final themes was reached. To become familiar with the data, all audio recordings were listened to, and the transcripts were read by two experienced qualitative researchers (LNJ and DG) as a first analytical step. Preliminary thoughts on the data were discussed in detail among the authors, who agreed on coping and adaptation as an analytical frame for the analysis. As a second step, all transcripts were then re-read by LNJ, who identified and coded meaning units connected to coping and adaptation. The codes were then categorized into 10 preliminary themes, and as the final step of the analysis, the data were reorganized according to the codes. Some themes were left out because they represented practical issues related to coping, such as hassles associated with getting the right equipment, which were not within the scope of the analysis. Eventually, the analysis resulted in the creation of four distinct analytical themes. A wide sample of illustrative quotes to support the analytical themes was selected by KZM and LNJ. All authors discussed and decided which of the quotes were most appropriate to use for underlining the findings within each theme.

During the last two interviews, no noteworthy new themes appeared, and the researchers agreed that data saturation had then been achieved.

## 3. Results

The study focused on the first month after discharge from the hospital. The families had been hospitalized for, on average, one week after receiving the diabetes diagnosis. Four overarching themes were identified as characteristic of how the families initially coped with and adapted to life with diabetes during the first few weeks.

**Figure 1:**
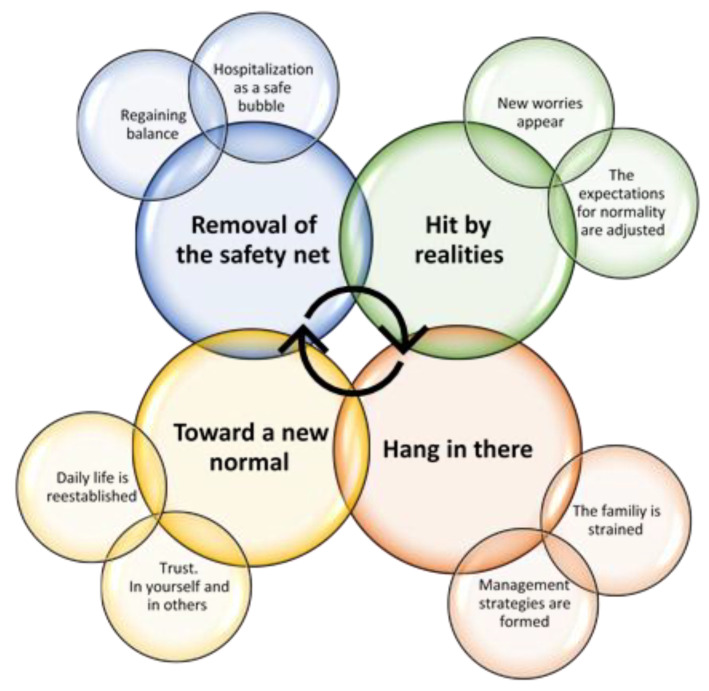
Overview of themes and subthemes identified in the interviews.

The themes and subthemes are presented in chronological order to underline the fact that the families are going through a process rather than being in a specific state. Not all parents go through all phases, as the processual elements overlap and different paths occur. However, the chronology of the themes, as reported below, was identifiable across interviews. Figure 1 provides an overview of the themes and subthemes.

### 3.1. Theme 1: Removal of the Safety Net

Across the interviews, parents described the transition from hospital to home as the time when their newly acquired diabetes management skills were put to the test. It was similar to having already learned to walk a tightrope, but now having to do it without a safety net. The first subtheme that emerged when the parents looked back at that time was: the parents described the in-patient period as ”a safe bubble”.

#### 3.1.1. Hospitalization as a Safe Bubble

*“It’s like, when you’re in the hospital, you live in some sort of strange bubble. Everything is a bit different, and you know that it will come to an end.”*

(parent of a 16-year-old)

A few families even compared the hospitalization period to a holiday, and one family referred to the hospital as a hotel because their daughter enjoyed eating the hospital food. The mother articulated her daughter’s thoughts:

*”It’s not as much fun to eat at home compared to eating at the hospital (laughs) Her father elaborated: “She lived on rolls and butter.”*

(Parents of a 6-year-old)

One characteristic of the safe bubble was that the families described the entire situation as profoundly different from their everyday lives. Not only was the new diagnosis different, but the surroundings, the daily routine, and the food had also suddenly changed. Thus, diabetes was just one part of this completely odd situation. The feeling of being in a safe bubble was reinforced by the feeling that help and support were available when the families needed them. The families did not feel left alone to handle the situation and experienced the HCPs as competent and caring, despite their busy schedule. Furthermore, the fact that the hospitalization had a clearly defined termination enhanced the bubble feeling. The bubble is here and now—the real world awaits outside, as described in the second subtheme: Regaining balance.

#### 3.1.2. Regaining Balance

The families reported that, during the hospitalization, they had looked forward to coming home. They had an expectation that everything would fall into place when they were back in their known environment. Quite a few parents had other children and, thus, needed to navigate hospitalization and normal daily life with school and leisure-time activities simultaneously. These families were especially eager to be discharged so they could reunite with their families. The ability to eat according to your own preferences was also frequently mentioned as something that would make everything easier outside the hospital. However, when the families eventually returned home, they realized they were now on their own.

*“Being in it alone is different, I’d say. Yes, it’s difficult when you’re in the middle of it and the rug is pulled out from under you. Will everyday life return?”*

(parent of a 11-year-old)

Quickly, the families realized that now—when everything else was normalized—the diabetes stood out. Their normal routines—sleep, food, school, work, leisure-time activities, travel, and social gatherings—were impacted and had to be altered to fit in with the new requirements of diabetes self-management. The expectation that everything would be easier at home was challenged. The hope of substantial improvement crumbled concurrently with the realization that diabetes had come to stay.

Differences in approaches to viewing and handling diabetes started to show. Some families rapidly accepted their new reality and worked their way around the challenges. These families were characterized by articulating that they would not allow diabetes to take up too much ”space” in their life.

*”But I think that we haven’t seen her getting diabetes as a major problem … I mean, I realize that it would have been easier without it and that the timing was bad… but getting diabetes is not the end of the world.”*

(parent of a 10-year-old)

On the other hand, families that seemed to struggle with adapting to their new situation seemed to have: (1) built up an unrealistic expectation that diabetes would not impact their life; and (2) more frequently articulated emotions such as anger, unfairness, or grief. Several of these families explained that some of the HCPs had unintentionally contributed to the illusion by promising that the child would be able to live as he or she used to, even with diabetes.

”I think we were encouraged to say ‘You can live as you used to.’ No we can’t! ‘Do they have a child with diabetes?’ Well, I never heard of any parent with a child with diabetes saying ‘well, we just live as we used to.’”

(parent of a 15-year-old)

## 3.2. Theme 2: Hit by the Realities

While regaining balance with diabetes as a new component to fit into an already busy family life, the parents slowly began to realize that this was now their reality. Some families explained that they had now proven they could handle the situation and were somehow waiting for the nightmare to pass. At this stage, the realization that diabetes had come to stay could feel similar to getting the diagnosis once again.

### 3.2.1. New Worries Appear

The foreseeable timeframe of hospitalization had come to an end, and with thoughts about the future and longer-term worries about the child’s life came the realization that there was nothing they could do about diabetes being a part of their lives forever. This feeling of forever was difficult for the families to handle when they tried to comprehend that they would never be able to take a break.

*”You can’t take a break from it […] and there is nothing you can do about it […] There are no easy fixes to it.”*

(parent of a 16-year-old)

When the parents felt they could handle the daily diabetes self-management and the immediate fear induced at the time of diagnosis started to decline, a new type of worry began to appear. This seemed to happen independently of where the family was in the process of adapting to the diagnosis. One common worry was the long-term consequences for the child in the years ahead. As one mother explained, those types of worries do not show up during hospitalization, which is dominated by worries about safety, e.g., hypo- and hyperglycemia.

*“You know… Thinking about how his childhood and youth will be with all this. All the problems that may arise and… But you don’t think about such things when you’re sitting in there [on the hospital ward].”*

(parent of a 3-year-old)

### 3.2.2. The Expectations for Normality Are Adjusted

The parents described how, during the first period at home, their expectations for normality had to be adjusted to suit their new reality. Many parents reported expecting to return to work, as well as to life as they knew it, as soon as possible.

*”I thought too quickly ’well, we just need to get back to normality. We’ll get him back to school, we’ll manage stuff’. Then it occurred to me that it can’t happen that fast.*

(parent of an 11-year-old)

Some parents revealed how, while they were hospitalized, they did not see any need to take leave from work, as recommended by the HCPs. However, when they understood the magnitude of the diabetes-related tasks, they conceded that taking leave from work was necessary if they were to adapt to their new situation. Furthermore, many of the families experienced that the state of emergency lasted much longer than they had expected it would. Some expected some kind of plateau to emerge and found it difficult when new challenges and tasks kept on coming. One parent explained that, two weeks after being discharged from the hospital, they were still in the process of ‘getting diabetes.’ This quote highlights the fact that the diabetes debut should be seen as a phase rather than a specific date.

*“When daily life is somehow reestablished and we’re back at work, I think you can begin to say that we are finished with the beginning. And you only have to do that once. That’s the best thing about the beginning. So, everything we’re learning now, we hopefully only have to learn once.”*

(parent of an 11-year-old)

For the reason that the state of emergency is longer than most families expect and the workload is constant and demanding, exhaustion starts to set in.

## 3.3. Theme 3: Hang in There

The long debut phase has consequences for the families. Although the families handle the pressure differently, any surplus energy begins to run out, and most parents feel exhausted. During this phase, the families report the importance of being able to hang in there. For some families, this ability seems to come more naturally than for other families. Especially families with less advantageous economic conditions or previous experience with chronic illness in the family demonstrated a remarkable perseverance. They might have gone through other difficult times and have experience with periods where you are not in control, but just have to go with the flow.

*“…it’s actually something we bear in mind from [older sisters’] diagnosis [of another chronic illness], which we got 2 years ago; remember, don’t think about all the ‘what-ifs’ in the future.”*

(parent of a 2-year-old)

### 3.3.1. The Family Is Strained

The realization of having reduced control for an indefinite period put a strain on the families. The learning curve kept increasing steeply, requiring their full attention. Furthermore, the lack of sleep was often mentioned as contributing to greater vulnerability within the family. Lack of sleep tended to be much more present in families where the child with diabetes was very young, whereas the older children, to a greater extent, dealt with low blood sugar during the night. Although the parents of older children did not necessarily have to get out of bed, they were still affected by lack of sleep because they were woken by the Continuous Glucose Monitor and stayed awake until they were assured their child had reacted appropriately to the alarm. One parent described his teenage daughter’s approach:

*”...and then she came shuffling in her pajamas to grab some chocolate milk and a piece of bread—and then she staggered back to bed.”*

(parent of a 17-year-old)

Furthermore, many families expressed feeling some degree of grief or guilt, which added to the intra-familial pressure. Some parents reported feeling responsible for their child getting diabetes, stating that this made them feel guilty. Others expressed feeling guilty about not having reacted to the symptoms earlier. In both cases, parents explained that although they were told at the hospital that they should not feel guilty, they still did. Aspects of grief were also frequently mentioned in relation to the present diabetes management and the expected impact that diabetes would have on their child’s life.

Grief about the long-term impact was especially present in families with previous knowledge about diabetes. If one of the parents or a close family member or friend had diabetes, they had an expectation of what awaited them, whereas families with no previous knowledge about diabetes did not know what to expect and therefore seemed to live in the moment without meeting the troubles halfway.

*”It hurts to watch him having all the red marks and adhesives on his tummy. He’s not that paunchy after all… I am not getting enough sleep… I am… [long pause] Sad about the trouble he will have because he has type 1 diabetes.”*

(Parent (with diabetes) of a 12-year-old)

The steep learning curve, sleep deprivation, and emotional strain meant that the families were extra exposed to intra-familial disagreements. Parents reported arguing about diabetes responsibilities. For example, they argued over who carried the heaviest burden and who had the right approach to self-management. Although they reported disagreements, most parents stressed that their good collaboration was key to staying on their feet during an intense period of family strain. They emphasized that having mutual respect for different ways of reacting and for different decisions from their own was important to getting through the crisis safely.

The tense situation also prompted disagreements between older children and their parents. The question of who was responsible for self-management and how much parents should be involved caused tension and snide remarks.

### 3.3.2. Management Strategies Are Formed

Being in a tense and difficult situation caused the parents to think of ways to navigate through the challenges. One frequently used coping method was to maintain or recover hope. Hope was often envisioned as a specific event that was expected to relieve the strain and make life easier. This light at the end of the tunnel gave the parents strength to keep on ”hanging in there”. An example of this was the expectation for the attachment of an insulin pump, which was expected to be

*“Something that will improve life, so you are a bit excited about when it will happen.”*

(parent of a 1-year-old)

Many parents reported being able to take one day at a time. One parent described how she had broken the learning process and stressful period down into more manageable pieces:

*“Well, I’m very aware of understanding a little bit at a time, although I knew a lot beforehand. Because… You shouldn’t begin to grasp the entire process. Just take one day at a time.”*

(parent of a 11-year-old)

As another way of managing the strains, many parents compared their situation to others. One parent described how seeing other families while they were hospitalized helped them put the diabetes diagnosis into perspective:

*“Of course, it could have been worse […] Well, although it sounds incredibly tough, I think that what saved us a lot was that we were on the pediatric hospital ward -and that some were worse off than us.”*

(Parent of a 1-year-old)

In addition to comparing diabetes to other conditions, as illustrated by the above quote, the parents also compared the age of their child to other ages. This went in both directions. Parents with younger children expressed as mitigating circumstances that their child was young at diagnosis because he or she would grow up with the diagnosis and they would be able to provide help and guidance for a longer period before the child had to undertake self-management tasks. As one of 5-year-old Noah’s parents explained, *”Yes, that’s something we’ve been telling ourselves; that he will never be able to remember that things have been different”.* In contrast, parents with older children expressed relief that their child was old enough to understand what was happening and had been diabetes free through part of childhood as 17-year-old Lilly’s parents explain:

*”It’s hard to compare her to a 5-year-old, because [at that age] you cannot sense yourself. So, in that respect, we’re lucky.”*

The third comparison strategy was seen in relation to the family’s resources. The parents compared their resources (mentally, economically, and socially) to those of other parents, who they expected would have more difficulties managing a pediatric diabetes diagnosis This was especially seen in families where the parent had a medium- or long-cycle higher education. One parent explained:

*“I’m thinking ‘we’re in a pretty good position, but there are parents who have lower IQs than we do, and then it‘s totally impossible to get an overview.”*

(parent of a 6-year-old)

## 3.4. Theme 4: Toward a New Normal

Although at the time of the interview the families were still at the very beginning of their process of adapting to a life with diabetes, most of the children had returned to school or daycare, as recommended by the HCPs at the hospital. Typically, one of the parents had returned to work, and normal family life with playdates, leisure activities, and birthday parties was back in full swing, now including a new important aspect: diabetes.

### 3.4.1. Trust: In Yourself and in Others

If you are to trust others with the responsibility for your child’s diabetes self-management, you first have to trust yourself. Some parents reported feeling insecure about managing their child’s diabetes. Parents of older children mentioned finding it difficult to balance the responsibilities between themselves and their child. However, most parents described how they made an effort to let the child manage as much as possible, although it was difficult for them to sit back and watch their child react differently to the measures than them. As one parent explained:

*“She’s very independent and… Usually she calculates correctly. Well, I’ve had to get used to the thought that; ‘Well, she won’t die. Even if that one [the CGM] beeps and things, she’s not going to die, right?”*

(parent of a 17-year-old)

For the reason that everyday life does not pause for the diabetes diagnosis, two weeks after having been discharged many parents had already experienced trust-related worries. Although the parents made a great effort to teach teachers and child caretakers the skills needed to support their child’s diabetes self-management, having to leave the responsibility for a child’s diabetes self-management to others required an enormous amount of trust. The potential consequences of insufficient insulin delivery are huge, and in combination with busy teacher schedules and the presence of many temporary staff, the parents of younger children were frequently physically present at their child’s daycare or school, while others were available on the telephone whenever the child was away. Support from the schools and teachers was seen as vital to the ability to trust others with the self-management of their child’s diabetes. Furthermore, because a child’s relational sphere reaches beyond school, there are numerous situations in which the child is left alone with diabetes self-management or is supervised by adults who know nothing about diabetes, e.g., during leisure-time activities or at friends’ houses. In the following quote, a parent expresses her concern about how she will be able to leave the responsibility to other parents.

”Because it is such a great responsibility and I have to leave that to other parents, who are not educated in the same way. Well, how?”

(parent of a 6-year-old)

### 3.4.2. Daily Life Is Reestablished

Realizing that diabetes has come to stay and that it comes with a substantial burden on the family, parents had begun considering how their lives could be altered to meet the requirements of diabetes management. Some parents had reconsidered their job situation, but the extent of those changes is illustrated in the following quote:

*”Our lives and the jobs we have and the way our life is organized at the moment—can it remain that way? Or should I find a job where I don’t have to show up before 9 AM? Because then we don’t have to leave her at the care facility [before school]… Well, there are several things that could be changed.”*

(parent of a 6-year-old)

In line with adjusting expectations for normality and viewing the time of diagnosis as a period rather than a specific date, the parents explained how new challenges kept emerging as the families gradually reestablished their daily lives. How do we manage diabetes during sports activities when the child might be on his or her own with a blood sugar level that is difficult to predict? How do we manage skiing holidays when the temperature is low, the food might be different from what the family typically eats, and the activity level is high? Experiencing normal activities such as sports and holidays—but now with diabetes—felt similar to experiencing the activity for the very first time. Some families were ready to face these challenges right from the beginning and had already been on a skiing holiday at the time of their interview. Others were more reluctant and expressed a need to get everyday life back on track before introducing new challenges. Especially for children aged 10 to 15, the pace of their reestablishment was not always aligned with that of their parents. Some children seemed to need a bit more time to adapt than their parents did. They felt that their parents kept going as if nothing had happened. In contrast, some children were ready long before their parents felt comfortable with it, as this parent of a 15-year-old described:

*“She has been really autonomous from day one. Actually, the doctors said to her on the third day: ‘Anna, just remember to get your parents on board’—And I can still feel that it’s out of my hands, because she wants to do it alone. She does it alone”.*

Regarding their future with diabetes, parents worry about their child. Parents who had previous experience with diabetes tended to report a less optimistic view of their future than did other parents because they were aware of the specific issues that may emerge, e.g., during adolescence or adulthood. However, most families reported having a bright view of their future. Many of them trusted that research on diabetes and available technologies would evolve to benefit their child, and some parents expressed hope for a cure. Generally, parents reported having faith in their ability to live well with their child’s diabetes.

*“I think things will have calmed down and that life seems more manageable [laughs]. I think that in one year, things will be good.”*

(parent of a 5-year-old)

## 4. Discussion

Although studies on the psychosocial aspects of parenting a child with diabetes do exist [7,12,15], few studies have provided qualitative insights into such an early stage in the adaptation process, thus beginning when a child is diagnosed with diabetes [16,17]. The present study generated unique insights into how parents cope and adjust during the very early stages after their child has been diagnosed, enabling us to present four overarching themes identified across all interviews: Theme 1: Removal of the safety net, describing how families, when discharged from the hospital, experience that they are now on their own. Theme 2: Hit by the realities, describing how parents realize the impact of living with diabetes. Theme 3: Hang in there, describing how families mobilize resources to get them through a tough period. Theme 4: Toward a new normal, describing how parents begin to reestablish and potentially rethink their lives with diabetes in mind. Across the four themes, a string of discussion points came up. Those points will be discussed below.

A serious chronic childhood illness is known to impact family functioning [18]. For parents of a child with diabetes, the initial phase following the diabetes diagnosis is associated with distress and even posttraumatic stress [7]. Across the interviews, it became clear that learning to cope with diabetes may be profoundly different from most other chronic childhood illnesses due to the coexistence of three components. The first component is the acute onset, which entails that all necessary changes are compressed into a short time, requiring the family to rapidly mobilize a set of crisis management skills. The second component is the steep learning curve occurring simultaneously and requiring levels of concentration and comprehension that may not be easily compatible with potential emotional imbalance. The third component, which seems to make adaptation to childhood diabetes stand out, is the constant and comprehensive self-management required. To keep their child alive, the parents must constantly be observing, measuring, calculating, making decisions about, and acting on the child’s blood sugar levels. The extent of this self-management regime leaves little time for rest, which is much needed if they are to recover and learn. In combination, the three components may require extraordinary coping skills and prolong the adaptation period.

Previous research has suggested that hope is a significant predictor of improved HbA1c [19]. One important finding in our study was the common use of hope as a coping strategy. Hope for the future was used as a reminder that the current situation is temporary and that things will get better. It was common for families to wait for some kind of plateau to appear that would provide a breathing space for them: going home to their safe environment to normalize the situation, a flattening of the learning curve to ease the cognitive load, a device to improve the sleep, time to settle, time to heal, etc. However, during their first month as parents of a child with diabetes, the plateau never seemed to appear, but in most cases, a new hope that there would be a new plateau was created. This finding is in accordance with previous research which, described adaptation to diabetes as an ongoing process because new issues to adjust to kept arising [16]. This constant adaptation underlines why the debut of childhood diabetes should be considered a process that stretches further than certainly the first month post-diagnosis.

Although hope can be used to help families navigate through a tough period, unrealistic hope induced by HCPs during hospitalization—e.g., that the child can live life as before—can harm the relationship and affect trust negatively. Therefore, it is important for HCPs to be aware of the fine line between inducing realistic and unrealistic hope.

In the literature, studies on the timing of the diabetes diagnosis in children have mainly been conducted retrospectively [8,20,21]. Investigating the newly diagnosed children a year after the date of diagnosis is very much in line with our acknowledgement of the prolonged debut period. However, all retrospective interview and survey studies entail the risk of significant recall bias because the parents have to rely on their memory, which may have been impacted by the nature of their current situation [22]. Interviewing the families as early as we did eliminated a substantial amount of the risk for recall bias.

We know that parents may experience grief related to their child’s diabetes diagnosis [23], and from our previous interview studies, we have learned that even after several years, parents become very emotional when describing the time when their child was diagnosed. We therefore expected that similar emotional reactions would occur during the interviews for the present study, but the experience of emotional burden was not as distinct as we expected it to be. We do not know why this was the case, but one potential explanation is that coping mechanisms cause the emotional response to be suppressed at this point in time, which is known as ‘repressive coping’ [24]. The parents spend so much energy learning about the biomedical aspects of diabetes, because this is necessary to keep their child alive, that little energy is left to attend to emotional difficulties. Our finding is that families with previous knowledge of diabetes do not view their future as brightly as do families with no previous knowledge of diabetes. This supports previous findings by Silverstein (2005), who underlined that parents’ personal experience with diabetes can lead to anxiety and depression because they know about acute and chronic complications [3]. This provides another potential explanation for the difference in descriptions of the time around the diagnosis. When parents are interviewed several years later, they have a better understanding of the implications the diabetes diagnosis has had for their family. This understanding may be comparable to the understanding that families with previous knowledge of diabetes already possess during the first month.

It is important to underline that all families cope with and adapt to their child’s diabetes diagnosis differently. Our study points to overarching themes that may or may not apply to the individual family. In general, all participating families seemed to have found a way to cope with their new situation.

### 4.1. Study Limitations

Inevitably, there are some limitations associated with the method of data collection. However, the present study adds to the existing body of knowledge by offering insights into the families’ experiences during a very early stage of adaptation to their new situation. We cannot rule out that a potential recruitment bias has entailed that families that struggled the most were not contacted by the nurses or were among the families that declined the invitation to participate after being contacted by telephone. For this reason, our result may not accurately represent adaptation and coping as experienced by all types of families. However, the choice of asking nurses to recruit the families was an essential and ethical one because it meant the families were approached by a familiar person and only contacted by the researcher if they agreed to participate.

### 4.2. Implications

The four overarching themes and subthemes can provide HCPs with knowledge about what the parents of children with newly diagnosed diabetes may experience during the first month after they are discharged from the hospital. For example, if HCPs are aware that they should not induce unrealistic hope about what to expect, they may be able to influence families’ expectations and hence avoid unnecessary disappointments.

The results may also guide the development of interventions to improve adaptation and the overall quality of life of families during the first month post-diagnosis.

Although we found signs of the themes occurring chronologically, more research on the same families is needed to investigate how their early coping and adaptation correlate with their progress later on. By conducting more interviews with the participating families, it will be possible to investigate how their adaptation and coping strategies change over time and, furthermore, how their views on living with diabetes evolve.

## 5. Conclusions

This study identified four overarching themes as characteristic of how the families initially cope with and adapt to life with diabetes during the first few weeks after their child’s diabetes diagnosis. Our findings indicate that parents’ educational level is not predictive of their ability to cope with and adapt to their childs’ diabetes diagnosis. Further, the findings underline that the diabetes debut should be seen as a phase rather than a specific date.

## Figures and Tables

**Table 1:** Characteristics of included families.

	Frequency	(%)
**Child age at discharge from hospital (years)**		
1–6	10	(50)
7–12	7	(35)
13–18	3	(15)
**Child gender**		
Female	10	(50)
Male	10	(50)
**Participating parents**		
Mother	5	(25)
Father	2	(10)
Mother and father	13	(65)
**Child participating in part of interview**		
Yes	9	(45)
No	11	(55)
**Mother educational level**		
Long-cycle higher education	9	(45)
Medium-cycle higher education	7	(35)
Vocational/upper secondary	2	(10)
Unknown	2	(10)
**Father educational level**		
Long-cycle higher education	8	(40)
Medium-cycle higher education	3	(15)
Vocational/upper secondary	5	(25)
Primary and lower secondary	2	(10)
Unknown	2	(10)
**Experience with diabetes in the family**		
Yes	3	(15)
No	17	(85)
**Civil status**		
Cohabiting parents	16	(80)
Divorced	3	(15)
Widowed	1	(5)
**Place of residence**		
Urban	5	(25)
Suburb	13	(65)
Rural	2	(10)

## Data Availability

The data presented in this study are available on request from the corresponding author.
